# Donor‐Specific Modeling of Alzheimer's Disease Progression via Cell‐Type‐Aware Single‐Cell Representations

**DOI:** 10.1002/alz70861_108378

**Published:** 2025-12-23

**Authors:** Hsin‐Yu Lai, Kyle J Travaglini, Andreas Tjaernberg, Anamika Agrawal, Eitan S Kaplan, Shubhabrata Mukherjee, Mariano I Gabitto

**Affiliations:** ^1^ Allen Institute for Brain Science, Seattle, WA USA; ^2^ University of Washington Alzheimer's Disease Research Center, University of Washington School of Medicine, Seattle, WA USA

## Abstract

**Background:**

Traditional approaches for studying cellular and molecular changes in Alzheimer's disease (AD) progression often rely on aggregated data across cell types, genes, and donor samples. These methods typically track population‐level changes in cells or genes across disease progression, which, although effective in identifying broad patterns, may obscure critical phenotypic variations. As a result, such models often fail to capture the nuanced trajectories of disease progression across individual donors.

**Method:**

We present a novel variational statistical framework that learns a per‐donor latent representation of disease progression from single‐cell transcriptomic data. Building upon existing platforms for the analysis of single‐cell data, we introduce a cell‐type‐aware, attention‐based architecture, which is invariant to the permutation of cells within a donor. To enhance interpretability, the latent representation is guided using downstream tasks, including neuropathological protein measurements (such as Aβ and *p* ‐tau density) and staging data.

**Result:**

We evaluated our model on two independent datasets—the Seattle Alzheimer’s Disease Cell Atlas (SEA‐AD) and the Religious Orders Study/Memory and Aging Project (ROSMAP)—focusing on brain regions such as the middle temporal gyrus and dorsolateral prefrontal cortex. Our approach successfully described the progression of AD along a multidimensional trajectory, inferred per‐donor pseudo‐progression scores, and predicted neuropathological information from scRNA‐seq data.

**Conclusion:**

By learning donor‐specific latent representations of AD progression, our model facilitates the investigation of relationships between molecular, cellular, and physiological changes throughout the disease course. This framework lays the groundwork for developing more personalized diagnostic and therapeutic strategies for Alzheimer’s disease.

